# Effects of Naming Language and Switch Predictability on Switch Costs in Bilingual Language Production

**DOI:** 10.3389/fpsyg.2018.00649

**Published:** 2018-05-23

**Authors:** Yueyue Liu, Song Chang, Li Li, Wenjuan Liu, Donggui Chen, Jinqiao Zhang, Ruiming Wang

**Affiliations:** ^1^Guangdong Provincial Key Laboratory of Mental Health and Cognitive Science, Center for Studies of Psychological Application, School of Psychology, South China Normal University, Guangzhou, China; ^2^School of Education, Qufu Normal University, Qufu, China; ^3^School of Educational Science, Ludong University, Yantai, China; ^4^The Key Laboratory of Chinese Learning and International Promotion, and College of International Culture, South China Normal University, Guangzhou, China; ^5^CAS Key Laboratory of Behavioral Science, Institute of Psychology, and Department of Psychology, University of Chinese Academy of Sciences, Beijing, China; ^6^College of Chinese Language and Culture, Jinan University, Guangzhou, China

**Keywords:** bilingual, language production, naming language, switch predictability, switch costs

## Abstract

Switch costs are defined as the phenomenon that bilinguals have worse performance in switch trials relative to non-switch trials. Bilinguals’ naming language and switch predictability have been found to influence the magnitude of switch costs. However, how these two factors modulate switch costs in different phases (i.e., lemma activation and language selection) during language production remains unclear. Most previous studies using the language switching paradigm did not dissociate lemma activation from language selection, because the language cue was either presented simultaneously with or prior to a stimulus. Therefore, here we modified the language switching paradigm by presenting a digit stimulus prior to a visual cue. This allowed us to dissociate lemma activation from language selection, and thus we were able to investigate the mechanisms underlying the effects of naming language and switch predictability on switch costs during the two different phases in language production. Unbalanced Indonesian-Chinese bilinguals were required to name digits in either their L1 (Indonesian) or L2 (Chinese), and their reaction times and electrophysiological responses were recorded. The behavioral results showed the effects of switch predictability on switch costs, with responses in switch trials being slower than those in non-switch trials in the low switch predictability condition, while there was no significant difference in response times between switch trials and non-switch trials in the high switch predictability condition. The event-related potential results showed that neither naming language nor switch predictability affected switch costs during the lemma activation phase, but both did so during the language selection phase, particularly at the language task schema competition stage. The results imply that naming language and switch predictability affect switch costs mainly during the language task schema competition stage.

## Introduction

Bilinguals have two language systems and thus face two options when they speak. However, they show remarkable flexibility and efficiency in switching from one language to another according to different interlocutors and varied scenarios during natural language production. In laboratory environments, language switching in production refers specifically to the process by which participants effectively switch from one language to another in accordance with continuously changing language cues. In general, longer reaction times (RTs) and higher error rates are obtained during language switching, these phenomena being known as switch costs. According to the inhibitory control (IC) model, switch costs are caused by the inhibition of the non-target language (Green, 1998). Specifically, as bilinguals have established two corresponding language task schemas to avoid confusion, they need to inhibit the non-target language to select the correct lemma; but when they switch to the formerly inhibited language, extra time is required to counter the inhibition and reactivate the language, leading to the switch costs (Green, 1998; Allport and Wylie, 2000). In the proactive interference model, one influential model derived from the task switching domain, switch costs are explained by assuming that the persistent activation of the previously used language causes either interference to the current language in switch trials or facilitation in non-switch trials (Allport et al., 1994; Green, 1998; Declerck et al., 2013). Moreover, bottom–up factors are also thought having possible influence on switch costs. For example, some aspects of Gollan and Ferreira’s (2009) findings implied an effect of lexical accessibility on switch costs. According to the bilingual interactive-activation (BIA) model, switch costs can arise from bottom-up activation of a given language node driven by presentation of a word in that language, which implies that the familiarity of L1 words also has an important effect on switch costs, since L1 lexical representations have higher resting level activations than L2 words (Grainger et al., 2010).

Meuter and Allport (1999) presented bilingual participants with a series of digits and required them to name digits in different languages according to a cue. Results showed larger switch costs into the more dominant language, which was known as asymmetric switch costs (Meuter and Allport, 1999). According to the IC model, asymmetrical switch costs suggest that non-proficient bilinguals suppress the more dominant L1 during L2 processing to a greater extent than vice versa. Consequently, switching into L1 from L2 involves more difficulty needed to overcome the inhibition of the L1 activation level on the previous trial than switching into L2 from L1 (Green, 1998). Although there are other interpretations of the mechanism underlying asymmetric switch costs (e.g., Bobb and Wodniecka, 2013; Declerck and Philipp, 2015; Khateb et al., 2017), increasing evidence from behavioral results (Meuter and Allport, 1999; Costa and Santesteban, 2004; Costa et al., 2006), functional neuroimaging studies (Abutalebi and Green, 2007, 2008; Wang et al., 2007; Garbin et al., 2011) and ERP studies (Jackson et al., 2001; Guo et al., 2013; Morales et al., 2015) lend some support to the inhibition perspective.

Previous studies have demonstrated that the relative proficiency of bilinguals’ naming languages mainly influence the magnitude of switch costs. As the IC model assumes: the more proficient the speaker is in the non-target language, the stronger inhibition, and thus the more effort required for reactivation. In other words, the dominant language among unbalanced bilinguals is more strongly inhibited than the weaker language. Therefore, it takes longer to overcome the initial inhibition. However, switch costs are similar in both switch directions among balanced bilinguals, whose non-native language has already reached a native-like level (Green, 1998; Philipp and Koch, 2009; Verhoef et al., 2009; Guo et al., 2013). Costa and Santesteban (2004) examined the role of naming language on switch costs. In their experiments, picture stimuli were presented simultaneously with language cues whereby participants were instructed to name the pictures in either their L1 or L2. They found larger switch costs in the L2-L1 than the L1-L2 switching direction among unbalanced bilinguals, whereas this difference disappeared among balanced bilinguals (Costa and Santesteban, 2004). Similarly, Costa et al. (2006) did not observe any difference between L1 and L2 switch costs among highly proficient bilinguals.

In recent years, researchers have used the event-related potentials (ERPs) technique to investigate the underlying mechanism of switch costs. Jackson et al. (2001) conducted an ERP study in which participants were instructed to name digits in their L1 or L2 in response to different background colors. Their results showed greater N2 amplitudes elicited by switch trials than by non-switch trials when naming in the L2, but no such difference in the L1, implying more inhibition of L1. Further ERP evidence for inhibition of the dominant language comes from Misra et al. (2012), who applied the blocked naming paradigm to investigate sustained language control. In their experiment, participants were required to name pictures in two successive blocks in one language and then the same pictures in a further two blocks in the other language. The repetition of pictures across blocks was expected to produce facilitation in the form of faster responses and more positive ERPs. However, they hypothesized that if both languages were activated when naming one language alone, there might be evidence of inhibition of the stronger L1 to enable naming in the weaker L2. Results showed no repetition advantage in the L2-L1 switch condition, which supported the inhibition of the L1 during bilingual language production. In addition, a significant priming effect was shown by identical pictures in the L1-L2 switch condition, which implied that the L2 did not require the same amount of inhibition as the L1. Taken together, the results of these researches confirm the proposal of the adaptive control hypothesis that bilinguals’ proficiency in the two languages is one important constraint on their ability to avoid switch costs (Green and Abutalebi, 2013).

Another crucial aspect to which researchers have gradually paid more attention is switch predictability (Macnamara et al., 1968; Declerck et al., 2013, 2015). Switch prediction is a mental process that allows bilinguals to predict the upcoming language according to the switch proportion across previous trials, and thus to anticipate the need for the same reaction or a different reaction. Researchers supporting predictability effects on language switch costs consider bilinguals as active predictors of the to-be-named language, rather than as passive processors. Such predictors can exploit knowledge about the to-be-produced response. Accurate switch prediction has appeared to facilitate the reaction, whereas inaccurate prediction has been shown to evoke greater switch costs. Macnamara et al. (1968) investigated the effects of switch predictability on language switching by contrasting performance in blocks with a predictable language sequence with that in an unpredictable language sequence. They found that, in comparison to an unpredictable sequence, a predictable sequence reduced switch costs, thereby demonstrating a predictability-based reduction in switch costs. In addition, Gollan and Ferreira (2009) investigated how voluntary-switching costs might differ from cued-switching costs. They found that, unlike cued switching, voluntary switching sometimes facilitated responses, and switch costs were not greater for the dominant language. Additionally, Gollan et al. (2014) contrasted cued switching versus voluntary switching to investigate switching efficiency, and found that voluntary responses were faster than cued responses on both switch and non-switch trials (Experiment 1), and that switch costs were smaller in the voluntary than the cued paradigm (Experiment 2). In other words, the participants were fully prepared, since they decided by themselves which language to use in each trial. A further insightful study conducted by Festman et al. (2010) explored switch costs during alternating language switching. Their results also showed reduced switch costs, when participants could predict the target language for each trial within the alternating switching context (Jackson et al., 2004; Festman et al., 2010).

Recent evidence for switch predictability effects on switch costs comes from Declerck et al. (2013). Declerck and colleagues introduced a sequence-based language switching paradigm, in which predictability of both language and the concept to be expressed could be exploited to prepare for an upcoming response. To ensure that participants exploited the predictable language sequence and the concept sequence, the amount of time available for preparation was manipulated. Their results showed that longer preparation for both language and concept led to smaller switch costs, implying a time-based preparation benefit. In order to systematically examine the influence of language and concept predictability, Declerck et al. (2015) combined sequence-based and cue-based language switching paradigms to further manipulate language predictability and concept predictability, respectively. Their results showed that the existence of a predictable response could reduce switch costs, whereas predictability of the language and the concept had only minor impacts on switch costs when they were examined independently.

All of these previous studies have contributed to our understanding of the effects of language proficiency and switch predictability on switch costs. However, how these two factors modulate switch costs in different phases during language production remains unclear. According to the language non-specific selection hypothesis (Green, 1998), lexical information in both target and non-target language is activated and competes to be produced against candidates within and across languages, and the most activated lexical item gets selected. Based on this hypothesis, bilingual language production consists of two main phases: lemma activation (translation-equivalent lemmas are activated in all potentially relevant languages by the stimulus) and language selection (the correct lemma is selected and the corresponding word is produced). However, manipulations in which the language cue and stimulus are presented simultaneously may bind the two phases together, and providing the cue prior to the stimulus might deny the opportunity to explore how bilinguals select the target language lemma after both are activated. The language switching tasks used in the above experiments could not appropriately dissociate these two phases of bilingual language production. Therefore, in order to dissociate lemma activation from language selection, here we adopted a modified language switching paradigm by presenting the cue after the stimulus. In the modified task, once the stimulus appeared (i.e., in the lemma activation phase), participants could activate the two corresponding language lemmas and then select the target language lemma, according to the following cue as soon as possible (i.e., in the language selection phase). In specific, language selection consists of the language task schema competition stage and the lemma selection stage (Green, 1998).

This modified language switching paradigm has been used in several previous studies (e.g., Chang et al., 2016; Ma et al., 2016; Khateb et al., 2017). In order to explore the locus of switch costs during bilingual language production, Chang et al. (2016) employed the ERPs technique and presented the cue and stimulus in two different presentation sequences (i.e., the stimulus-cue sequence and the cue-stimulus sequence). Particularly, in the stimulus-cue sequence, they found reversed switch costs as early as 220 ms after the cue onset, whereas switch costs in L1 occurred after 350–500 ms post-cue onset, suggesting that switch costs mainly occurred at the lemma selection stage. Ma et al. (2016) examined the effect of the postcuing manipulation comparing short and long Stimulus-Cue intervals (SCIs) in a language-switching digit-naming task. They found significant switch costs with postcuing, which support the notion of persisting activation from a recently used language, even at the level of specific lemmas. Recently, Khateb et al. (2017) examined the interplay between global and local processes in bilingual language control with cued picture naming. Global control refers to the activation and inhibition at the level of the language schema, while local control means the local lateral connections between translation- equivalent lemmas within the bilingual lexicon. The language cue could precede the picture, follow it, or appear simultaneously with it. Particularly, the postcuing manipulation demonstrated that persisting language schema activation was equal for both languages of unbalanced bilinguals even after specific translation-equivalent lemmas had been selected, and that lemma selection among two activated translation equivalents was again equal for L1 and L2 for local control.

In the current study, we adopted this modified language switching paradigm to investigate the mechanisms underlying the effects of naming language and switch predictability on switch costs during the different phases in language production. That is, how these two factors modulate switch costs in different phases (i.e., lemma activation and language selection) during language production. Furthermore, switch predictability was manipulated by implementing two experimental sequences with 30 and 70% of switch trials, respectively, to form a low switch predictability condition and a high switch predictability condition. Before the experiment, participants were informed about the different switch probabilities, thus they could make an educated guess whether the following trial would be in the same language or not. For example, in the low switch predictability condition, there were far fewer switch trials than non-switch trials. In this sequence, participants could recognize the clear difference between the two kinds of trials, and would thus achieve high predictability for non-switch trials and low predictability for switch trials. We employed the ERPs technique to tap into the language activation phase, when no behavioral response could be measured. ERPs help to elucidate the time-course of cognitive mechanisms involved in bilingual language production. The examination of ERPs time-locked to stimulus and to cues could be informative for the effects of naming language and switch predictability on switch costs, respectively.

## Materials and Methods

### Participants

Twenty Indonesian-Chinese bilinguals (5 males, 15 females, 22 ± 3.30 years of age) participated in the present study. All were Indonesian native speakers and began to learn Chinese after the age of 9 (i.e., late bilinguals). They first came to China at the age of 18.1 (*SD* = 1.05), and had resided in China for 45.5 months (*SD* = 8.71) at the time of experiments. Besides, they mainly spoke Indonesian (L1) before coming to China, and mainly spoke Chinese (L2) when in China. Participants were recruited according to the criteria that Indonesian was their first language and that they felt able to name the digits 1 to 8 fluently in Chinese. Their self-assessed language proficiency ratings were based on a scale of 0–10, in which 10 indicated the highest level of proficiency. The average proficiency rating in Indonesian (their L1) was 8.16 (*SD* = 1.05), and in Chinese (their L2) 6.63 (*SD* = 1.05). The *t*-test comparing L1 and L2 scores imply that participants are more dominant in their native language [*t*(19) = 4.67, *p* < 0.001]. They all had normal or corrected-to-normal vision. Each participant was offered a small monetary reimbursement for participating in the study. All participants gave written informed consent in accordance with the Declaration of Helsinki. The study was approved by the Ethics Review Board of School of Psychology, South China Normal University.

### Materials

In the current study, the participants performed a speeded digit-naming task in which they repeatedly named the Arabic digits (1–8) in their L1 or L2, according to the cue color (blue or red). Blue and red squares were presented pseudo-randomly so that subsequent trials could require the use of either the same language or a different one. In this way, four different language transition conditions were obtained: L1 non-switch trials, L1-L2 switch trials, L2-L1 switch trials, and L2 non-switch trials. The two different switch proportions (30% switch proportion and 70% switch proportion) were manipulated, forming low switch predictability (30%) and high switch predictability (70%) sequences. Each switch predictability sequence had 240 trials (comprising half L1 and half L2 trials). Experimental materials were matched into 4 lists in order to counterbalance the color cues for language naming and switch predictability sequences. Red cue for Chinese, blue cue for Indonesian, low switch predictability first, and then high switch predictability in list 1; Red cue for Chinese, blue for Indonesian, high switch first, and then low switch in list 2; Red for Indonesian, blue for Chinese, low switch first and then high switch in list 3; Red for Indonesian, blue for Chinese, high switch first and then low switch in list 4. Twenty participants were randomly divided into 4 groups for 4 lists of materials.

### Procedure

**FIGURE 1:**
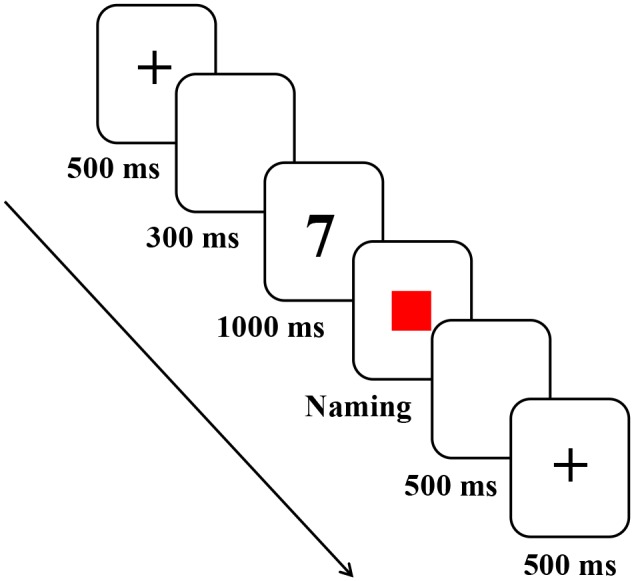
Example of a trial sequence.

Prior to the experiment, the instructions were presented to participants both orally and visually, and participants were informed about the different switch probabilities, with an emphasis on both speed and accuracy. In the low switch predictability sequence, each trial began with the presentation of a fixation cross for 500 ms followed by a blank screen for 300 ms. Each digit was displayed for 1000 ms and then it was removed from the screen when the cue (width = 10%, height = 15% in E-prime) was presented. The cue remained on the screen until the participant responded. Participants were required to name the digit as quickly and accurately as possible in either their L1 or L2 according to the color cue. There was a 500 ms interval between trials. In the high switch predictability sequence, the naming task was similar to that in the low switch predictability sequence, except for the proportion of switch trials. Participants completed the two tasks with the different proportion of switch trials. The assignment of the two switch predictability sequences and the color cues for L1 and L2 responses were counterbalanced across participants. The experiment consisted of a total of 480 stimuli. An example trial sequence is illustrated in **Figure 1**.

### Electrophysiological Recording and Analysis

ERPs were continuously sampled at 1000 Hz by a 32-channel Quik cap (NeuroScan Inc.) with a band pass filter of 0.05 and 70 Hz, online referenced to the left mastoid. Thirty-three Ag/AgCl electrodes were placed according to the 10–20 convention (FP1, FP2, F3, Fz, F4, F7, F8, FT7, FT8, FC3, FCz, FC4, T7, T8, C3, Cz, C4, TP7, TP8, CP3, CPz, CP4, TP7, TP8, P3, Pz, P4, P7, P8, O1, Oz, O2, right mastoid). In addition, horizontal electro-oculogram (EOG) was recorded by placing two electrodes on the outer canthus of each eye, and vertical EOG by positioning two electrodes above and below the left eye for artifact rejection purposes. Electrode impedances were kept below 5 kΩ. ERPs were digitally filtered at a low-pass of 30 Hz (24 dB setting). Naming latencies were usually longer than 500 ms after the stimulus onset. Therefore, epochs ranging from -100 ms to 500 ms after stimulus onset were used to avoid the muscle artifact induced by naming. Epochs with voltages exceeding ± 100 μV in the EEG were rejected. Only trials free from eye and muscle artifacts were included in the averages. Following these criteria, no less than 30 trials were averaged in each condition. The individual ERPs were then grand averaged for presentation.

Based on visual inspection and previous studies (Jackson et al., 2001; Christoffels et al., 2007; Martin et al., 2009; Verhoef et al., 2010; Chen et al., 2015), two time windows were chosen for stimulus-locked analysis, namely 180–280 ms and 350–500 ms post-stimulus onset. A further two time windows were selected for analysis of the mean amplitudes of components of interest for cue-locked ERPs, namely 200–300 ms and 350–500 ms post-cue onset. Consistent with previous studies (Kroll et al., 2008; Zhang et al., 2012), we quantified N2 by a mean amplitude measure for three frontal electrodes (F3, Fz, F4), three central electrodes (C3, Cz, C4), and three parietal electrodes (P3, Pz, P4). And a 2 (naming language: Indonesian or Chinese) ^∗^ 2 (switch predictability: low switch predictability or high switch predictability) ^∗^ 2 (language transition: switch or non-switch) ^∗^ 9 (electrodes) repeated measures ANOVA was performed for the mean amplitudes of ERPs in each time window. The results showed that three factors interact with electrodes; further analysis found that the effect of N2 is mainly manifested over electrodes F3, Fz, F4. In previous studies, N2 was observed to be maximal over the frontal and central scalp. Moreover, the frontal cortex is related to general executive functions such as response switching and response suppression (Dove et al., 2000; Sohn et al., 2000; Konishi et al., 2003). This view is supported by neuroimaging studies that yielded enhanced activation of the dorsolateral pre-frontal cortex during language switching (Hernandez et al., 2000, 2001). Therefore, based on the topographical distribution of the effects we were interested in, we chose prefrontal electrodes (i.e., F3, Fz, F4) to calculate the N2 amplitude. Similarly, consistent with previous studies (Martin et al., 2009; Chen et al., 2015), we quantified N400 by a mean amplitude measure for three frontal F3, Fz, F4, three central C3, Cz, C4, and three parietal P3, Pz, P4. And a 2 (naming language: Indonesian or Chinese) ^∗^ 2 (switch predictability: low switch predictability or high switch predictability) ^∗^ 2 (language transition: switch or non-switch) ^∗^ 9 (electrodes) repeated measures ANOVA was performed for the mean amplitudes of ERPs in each time window. The results showed that three factors interact with electrodes; further analysis found that the effect of N400 is mainly manifested over electrodes C3, Cz, C4, P3, Pz, P4. Moreover, N400 was maximal over centroparietal region (Martin et al., 2009). Therefore, based on the topographical distribution of the effects we were interested in, we chose centroparietal electrodes (i.e., C3, Cz, C4, P3, Pz, P4) to calculate the N400 amplitude.

Separate analyses were conducted for prefrontal and middle-lateral electrode sites. Three prefrontal electrodes (F3, Fz, and F4) and six middle-lateral electrodes (C3, Cz, C4, P3, Pz, and P4) were selected as important electrode sites (Folstein and Van Petten, 2008; Kutas and Federmeier, 2011). A 2 (naming language: Indonesian or Chinese) ^∗^ 2 (switch predictability: low switch predictability or high switch predictability) ^∗^ 2 (language transition: switch or non-switch) repeated measures ANOVA was performed on the mean amplitudes of ERPs in each time window. Geisser–Greenhouse corrections were reported when applicable, but unadjusted degrees of freedom were presented.

## Results

### Behavioral Data

**FIGURE 2:**
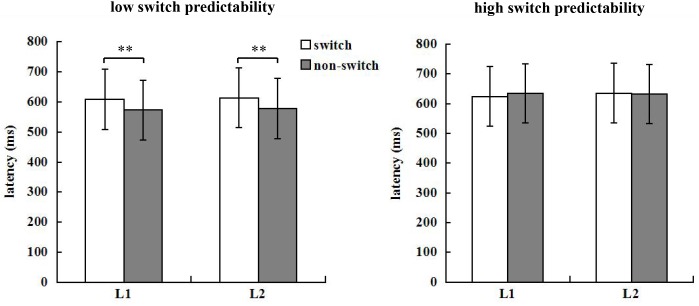
Mean RTs across different conditions for the low and high switch predictability condition.

The cleaning-up procedure in Guo et al. (2013) was adopted. In RT analysis, correct responses made without incorrect naming or verbal disfluency were included. RTs for correct naming trials shorter than 200 ms and longer than 2000 ms were exclude as outliers, and a secondary trimming step was used to exclude naming latencies 2.5 standard deviations above or below each individual’s mean value. This procedure led to the exclusion of 3.75% of correct naming trials as outliers across all behavioral conditions. All participants performed with high accuracy (above 0.98) and there were no significant main effects or interactions. Only the mean RT was calculated for each of the eight conditions, as summarized in **Figure 2**.

As mentioned above, we performed a 2 ^∗^ 2 ^∗^ 2 ANOVA for the RT data. The RT analysis revealed that the main effect of naming language was not significant, *F*(1,19) = 0.26, *p* = 0.613, ${\text{η}}_{\text{p}}^{2}$ = 0.014. The main effect of switch predictability was significant, *F*(1,19) = 20.12, *p* < 0.001, ${\text{η}}_{\text{p}}^{2}$ = 0.514. Responses in the high switch predictability condition (*M* = 631.53 ms) were slower than those in the low switch predictability condition (*M* = 593.42 ms). The main effect of language transition was also significant, *F*(1,19) = 6.61, *p* = 0.019, ${\text{η}}_{\text{p}}^{2}$ = 0.258. Responses in switch trials (*M* = 620.55 ms) were slower than those in non-switch trials (*M* = 604.40 ms), indicating 16.5 ms of language switch costs.

The interaction between naming language and language transition was not significant, *F*(1,19) = 0.68, *p* = 0.420, ${\text{η}}_{\text{p}}^{2}$ = 0.035. The interaction between naming language and switch predictability was also not significant, *F*(1,19) *<* 0.001, *p* = 0.970, ${\text{η}}_{\text{p}}^{2}$* <* 0.001, whereas that between switch predictability and language transition was significant, *F*(1,19) = 22.27, *p* < 0.001, ${\text{η}}_{\text{p}}^{2}$ = 0.540. Further simple main effect analysis showed that responses in switch trials (*M* = 611.40 ms) were slower than those in non-switch trials (*M* = 575.44 ms) in the low switch predictability condition, *F*(1,19) = 16.20, *p <* 0.001, ${\text{η}}_{\text{p}}^{2}$ = 0.460. In contrast, in the high switch predictability condition, there was no significant RT difference between switch trials and non-switch trials, *F*(1,19) = 0.39, *p* = 0.538, ${\text{η}}_{\text{p}}^{2}$ = 0.020. The three-way interaction between naming language, switch predictability, and language transition did not reach significance, *F*(1,19) = 1.23, *p* = 0.280, ${\text{η}}_{\text{p}}^{2}$ = 0.061.

### Linear Mixed Effect Analysis

Since we used different digits and did not include digit as a source of variability in the statistical analyses of ANOVA, we switched to linear mixed effects (LME) models to accommodate crossed random effects, which could also allow us to take into account the imbalance in the number of trials that feed into different conditions. The linear mixed effects modeling (the lme4 package, version 1.1.13) was employed to analyze response times and accuracy rates. Following the recent suggestions (Bates et al., 2015; Matuschek et al., 2017), we determined the best fitting random effect structure using forward model comparison (the use of backward model comparison would often result in non-convergences). We built a base model with random participants and item intercepts, in addition to all the fixed effects: naming language (L1:L2), switch predictability (low switch predictability: high switch predictability), language transition (non-switch: switch), all contrast-coded, and their interactions. A random slope was kept in the random effect structure if its addition would increase the model fit (to avoid anti-conservativeness, following Matuschek et al., 2017, the alpha level was set to 0.2 instead of 0.05). The *df* and the *p*-values were computed using the lmerTest package (Kuznetsova et al., 2015).

Models for RTs are shown in **Table 1**. There was a significant effect of switch predictability, showing that responses in the high switch predictability condition were slower than those in the low switch predictability condition. The effect of language transition was also significant, showing that responses in switch trials were slower than those in non-switch trials. Naming language did not produce a significant effect. The interaction between switch predictability and language transition reached significance, showing that responses in switch trials were slower than those in non-switch trials in the low switch predictability condition, while in the high switch predictability condition, there was no significant RT difference between switch trials and non-switch trials.

**Table 1:** Results of LME on RT.

	Estimate	*SE*	*df*	*t*	*p*
Intercept	622.4285	23.8073	21	26.144	<0.001
Naming Language	-3.4590	5.2889	21	-0.654	0.5200
Switch Predictability	-29.3551	4.5687	19	-6.425	<0.001
Language Transition	7.9030	3.2475	19	2.434	<0.05
Naming Language: Language Transition	0.5879	3.4508	6	0.170	0.8703
Switch Predictability: Language Transition	8.0159	1.9983	6444	4.011	<0.001
Naming Language: Switch Predictability	0.3072	2.5005	10	0.123	0.9046
Naming Language: Switch Predictability: Language Transition	0.2691	2.1015	78	0.128	0.8984

*The final LME model included, apart from the random participant and item intercepts, random participant slopes for Naming Language, Switch Predictability, Language Transition and the interaction between Naming Language and Switch Predictability and random item slopes for Naming Language, the interaction between Naming Language and Switch Predictability and the interaction between Naming Language and Language Transition. RT = lmer [RT∼ cNaming Language ^∗^ cSwitch Predictability ^∗^ cLanguage Transition + (cNaming Language + cSwitch Predictability + cLanguage Transition + cNaming Language: cSwitch Predictability: cLanguage Transition + 1| Subject) + (cNaming Language + cNaming Language: cSwitch Predictability + cNaming Language: cLanguage Transition + 1| Digit), RT, control = lmerControl (optimizer = “bobyqa”, optCtrl = list (maxfun = 1e6))].*

Models for accuracy (ACC) are shown in **Table 2**. Switch predictability produced a marginally significant effect, showing participants made more errors under the high switch predictability condition than the low switch predictability condition. Both the effects of naming language and language transition were not significant. The interaction between naming language and language transition reached significance, indicating that participants made more errors in switch trials compared to non-switch trials in L2, but no such difference in L1. The interaction between naming language and switch predictability also reached significance, with more errors under the high switch predictability condition than the low switch predictability condition in L2, while no such difference in L1, suggesting that switch predictability had larger effect on L2. Other interaction effects did not reach significance.

**Table 2:** Results of LME on ACC.

	Estimate	*SE*	*Z*	*P*
Intercept	3.298899	0.213537	15.449	<0.001
Naming Language	0.054823	0.077810	0.705	0.481078
Switch Predictability	0.158052	0.088607	1.784	0.074465
Language Transition	-0.031955	0.052974	-0.603	0.546365
Naming Language: Language Transition	0.126396	0.053046	2.383	<0.05
Switch Predictability: Language Transition	0.002224	0.053328	0.042	0.966732
Naming Language: Switch Predictability	0.182143	0.055076	3.307	<0.001
Naming Language: Switch Predictability: Language Transition	0.027063	0.053136	0.509	0.610536

*The final LME model included, apart from the random participant and item intercepts, random participant slopes for Naming Language and Switch Predictability. ACC = glmer [ACC∼ cNaming Language ^∗^ cSwitch Predictability ^∗^ cLanguage Transition + (cNaming Language + cSwitch Predictability + 1| Subject) + (1| Digit), ACC, family = “binomial”, control = glmerControl (optimizer = “bobyqa”, optCtrl = list (maxfun = 1e6))].*

In conclusion, the primary aim of our research was to test the effects of naming language and switch predictability on switch costs. Our LME results were consistent with the effects of the ANOVA data, demonstrating that the digit-item did not modulate the current pattern of ANOVA data.

### Electrophysiological Data

#### Digit-Locked Electrophysiological Data

**FIGURE 3:**
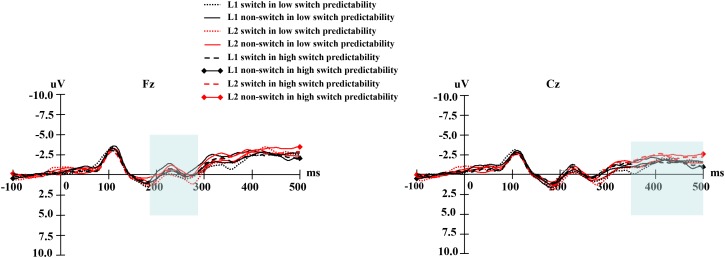
**(Left)** Shows the averaged the early phase and **(right)** the averaged the late phase elicited by digits.

For digit-locked grand average waves, at the early phase, there was a negative-going component that peaked at approximately 230 ms and lasted approximately 100 ms after stimuli onset. At the late phase, digits elicited a negative-going wave, which began around 350 ms and lasted until 500 ms after stimuli onset. **Table 3** shows the mean amplitudes and standard errors of the early phase elicited by digits, and **Table 4** shows the mean amplitudes and standard errors of the late phase elicited by digits. **Figure 3** shows the averaged early and late phases elicited by the digits. For both 180–280 ms and 350–500 ms windows, the ANOVA performed over the frontal and middle-lateral electrodes showed no significant main effects or interactions after digits onset.

**Table 3:** Mean amplitudes and standard errors of the early phase elicited by digits.

	Low switch predictability		High switch predictability	
	Switch	Non-switch	Switch	Non-switch
L1	0.57 ± 3.20	0.22 ± 3.37	0.19 ± 2.16	-0.40 ± 2.77
L2	0.61 ± 3.16	0.01 ± 2.45	0.26 ± 2.79	0.06 ± 2.46

**Table 4:** Mean amplitudes and standard errors of the late phase elicited by digits.

	Low switch predictability		High switch predictability	
	Switch	Non-switch	Switch	Non-switch
L1	0.25 ± 2.39	-0.37 ± 4.05	0.43 ± 2.15	-0.28 ± 2.85
L2	-0.12 ± 2.63	-0.05 ± 3.03	-0.71 ± 3.23	-0.34 ± 2.51

#### Cue-Locked Electrophysiological Data

**FIGURE 4:**
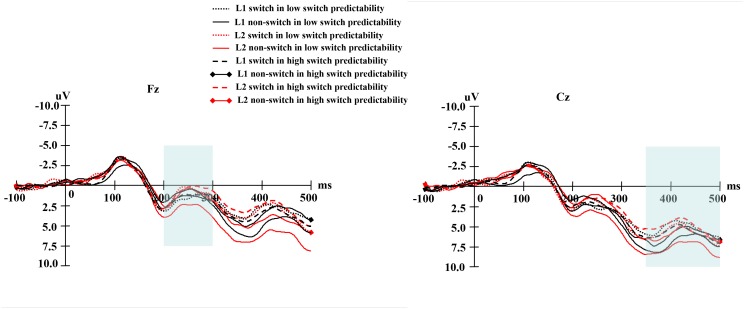
**(Left)** Shows the averaged the early phase and **(right)** the averaged the late phase elicited by cues.

For cue-locked grand average waves, at the early phase, there was a negative-going wave that peaked at approximately 250 ms and lasted approximately 100 ms after cues onset. At the late phase, cues elicited a negative-going component, which began around 350 ms and lasted until 500 ms after cues onset. **Table 5** shows the mean amplitudes and standard errors of the early phase elicited by cues, and **Table 6** shows the mean amplitudes and standard errors of the late phase elicited by cues. **Figure 4** shows the averaged early and late phases elicited by cues. The cue-locked electrophysiological data are discussed further below in terms of the early vs. late phases of word selection.

**Table 5:** Mean amplitudes and standard errors of the early phase elicited by cues.

	Low switch predictability		High switch predictability	
	Switch	Non-switch	Switch	Non-switch
L1	2.00 ± 2.51	1.59 ± 4.23	1.88 ± 2.73	2.00 ± 3.07
L2	1.36 ± 2.98	3.22 ± 3.65	1.17 ± 2.79	1.55 ± 2.89

**Table 6:** Mean amplitudes and standard errors of the late phase elicited by cues.

	Low switch predictability		High switch predictability	
	Switch	Non-switch	Switch	Non-switch
L1	6.39 ± 3.72	7.38 ± 5.53	6.54 ± 2.28	6.47 ± 3.02
L2	7.07 ± 4.23	8.32 ± 4.00	5.77 ± 3.27	6.68 ± 3.71

**FIGURE 5:**
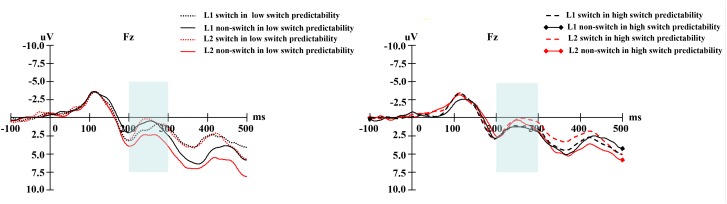
**(Left)** For the low switch predictability condition, the grand average of the early phase elicited by language cues for naming digits in L1 and L2 at the Fz electrode. **(Right)** For the high switch predictability condition, the grand average of the early phase elicited by language cues for naming digits in L1 and L2 at the Fz electrode.

**FIGURE 6:**
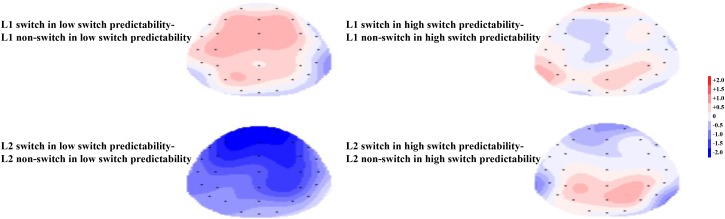
**(Left)** For the low switch predictability condition, the topographical maps of the early phase for the distribution of the difference between the same language in different transition trials, i.e., switch trials minus non-switch trials. **(Right)** For the high switch predictability condition, the topographical maps of the early phase for the distribution of the difference between the same language in different transition trials, i.e., switch trials minus non-switch trials.

In terms of the cue-locked electrophysiological data at the early phase of language selection (see **Figure 5**), an ANOVA on the data from the pre-frontal electrodes showed no significant main effects of naming language, switch predictability, or language transition. However, the interaction between naming language and language transition reached significance, *F*(1,19) = 5.80, *p* = 0.026, ${\text{η}}_{\text{p}}^{2}$ = 0.234. Further simple main effect analysis showed a larger negative-going wave elicited by switch trials compared to non-switch trials in L2, *F*(1,19) = 7.50, *p* = 0.013, ${\text{η}}_{\text{p}}^{2}$ = 0.283, but no such difference was observed in L1, *F*(1,19) = 0.10, *p* = 0.757, ${\text{η}}_{\text{p}}^{2}$ = 0.005. This finding indicates that greater inhibition of L1 is required to access L2, which is in line with the results of previous studies (e.g., Jackson et al., 2001). The interaction between switch predictability and language transition was insignificant, *F*(1,19) = 0.52, *p* = 0.476, ${\text{η}}_{\text{p}}^{2}$ = 0.027. The interaction between naming language and switch predictability was marginally significant, *F*(1,19) = 3.27, *p* = 0.086, ${\text{η}}_{\text{p}}^{2}$ = 0.147. Furthermore, there was significant interaction between naming language, switch predictability, and language transition, *F*(1,19) = 6.73, *p* = 0.018, ${\text{η}}_{\text{p}}^{2}$ = 0.262. Further simple main effect analysis showed a more negative amplitude for switch trials than for non-switch trials in L2 under the low switch predictability condition, *F*(1,19) = 10.04, *p* = 0.005, ${\text{η}}_{\text{p}}^{2}$ = 0.346, whereas this difference was not significant in either language under the high switch predictability condition (*ps* > 0.10). This finding suggests that the effects of naming language on switch costs were susceptible to switch predictability. The topographical maps of the early phase for the distribution of the difference between the same language in different transition trials are illustrated in **Figure 6**.

**FIGURE 7:**
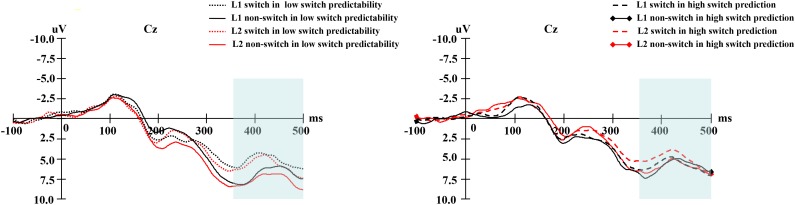
**(Left)** For the low switch predictability condition, the grand average of the late phase elicited by language cues for naming digits in L1 and L2 at the Cz electrode. **(Right)** For the high switch predictability condition, the grand average of the late phase elicited by language cues for naming digits in L1 and L2 at the Cz electrode.

In terms of the cue-locked electrophysiological data at the late phase of language selection (see **Figure 7**), the ANOVA on the data from the middle-lateral electrodes showed that the main effect of naming language was not significant, *F*(1,19) = 0.97, *p* = 0.336, ${\text{η}}_{\text{p}}^{2}$ = 0.049. However, a marginally significant main effect of switch predictability was obtained, *F*(1,19) = 3.06, *p* = 0.096, ${\text{η}}_{\text{p}}^{2}$ = 0.139. In addition, there was also a significant main effect of language transition, *F*(1,19) = 5.03, *p* = 0.037, ${\text{η}}_{\text{p}}^{2}$ = 0.210, with a bigger negative-going wave elicited by switch trials than by non-switch trials, suggesting greater inhibition during the switch process than the non-switch process.

The interaction between naming language and language transition was not significant, *F*(1,19) = 1.25, *p* = 0.276, ${\text{η}}_{\text{p}}^{2}$ = 0.062. Similarly, the interaction between switch predictability and language transition was insignificant, *F*(1,19) = 1.13, *p* = 0.299, ${\text{η}}_{\text{p}}^{2}$ = 0.057. In addition, naming language marginally interacted with switch predictability, *F*(1,19) = 3.30, *p* = 0.085, ${\text{η}}_{\text{p}}^{2}$ = 0.148.

**FIGURE 8:**
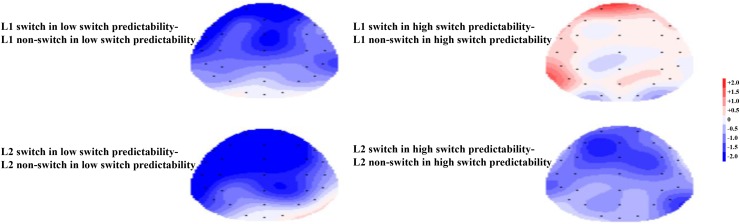
**(Left)** For the low switch predictability condition, the topographical maps of the late phase for the distribution of the difference between the same language in different transition trials, i.e., switch trials minus non-switch trials. **(Right)** For the high switch predictability condition, the topographical maps of the late phase for the distribution of the difference between the same language in different transition trials, i.e., switch trials minus non-switch trials.

Finally, we found no significant interaction effect between naming language, switch predictability, and language transition, *F*(1,19) = 0.32, *p* = 0.577, ${\text{η}}_{\text{p}}^{2}$ = 0.017. The topographical maps of the late phase for the distribution of the difference between the same language in different transition trials are illustrated in **Figure 8**.

## Discussion

In the current study, we set out to explore the effects of naming language and switch predictability on switch costs during two phases of bilingual language production, namely the lemma activation phase and the language selection phase. A modified language switching paradigm was implemented to dissociate lemma activation from language selection by presenting stimuli prior to visual cues for naming language. The results are discussed in light of the IC model and the proactive interference model.

### Factors Influencing Switch Costs

The behavioral results showed evidence of switch costs, while no asymmetry of switch costs was observed for unbalanced bilinguals. The lack of an asymmetry was perhaps not that surprising, given the fact that participants had been staying in a Chinese-speaking context for a long period (about 4 years) at the time of experiment, and so perhaps were not so unbalanced as the proficiency scores suggested.

Two main findings about the effects of naming language and switch predictability on switch costs were revealed from the present data. First, the RT measures showed no main effect of naming language on switch costs, but the ERP data revealed switch costs in L2 only. This discrepancy between behavioral and ERP results indicates the existence of proficiency difference in unbalanced bilinguals, which is likely to be detected via the more sensitive technology. The ERP results are in line with previous studies. For example, Jackson et al. (2001) required unbalanced bilinguals to complete a digit-naming task with cues, and also found switch trials elicited greater negative N2 amplitudes than non-switch trials did when naming in L2, whereas the difference was not significant when naming in L1. Results in the current study supported the assumption of the IC model that more inhibition was required to suppress L1 on L1-L2 switching direction trials than to suppress L2 on L2-L1 switching direction trials. The fact that the amplitude of the N2 was larger when switching into L2 but did not differ between switch and non-switch trials in L1 may indicate that rather than targeted trial-by-trial inhibition of L1, participants just applied more sustained inhibition to L1 throughout the task. Misra et al. (2012) applied the blocked naming paradigm to explore the inhibition of the native language. Their ERP data showed greater negativity associated with the L1 when it followed the L2 endured beyond the immediate switch of language, implying long-lasting inhibition of the L1. This would explain why inhibition levels were equivalent on L1 switch and non-switch trials, as they were all equally inhibited. Still, participants might have needed to exert additional inhibition when trying to select L2 after an L1 trial, because bottom-up factors (the recent experience of naming in L1, and listening to an L1 word) would have temporarily boosted L1 activation and increased interference with L2 selection. Hence, our results provided additional evidence for bottom-up factors’ influence on language switch costs (see also Gollan and Ferreira, 2009; Grainger et al., 2010).

A second main finding is that we observed a reliable effect of switch predictability on switch costs. The behavioral results showed that switch costs only appeared in the low switch predictability condition, with no switch costs in the high switch predictability condition. Consistent with this behavioral data, the ERP measures showed that greater negative ERP components were elicited by switch trials than non-switch trials only in the low switch predictability condition, and there was no such difference in the high switch predictability condition. A possible reason for these findings is that, there were far fewer switch trials than non-switch trials in the low switch predictability condition. In this condition, participants achieved high predictability for non-switch trials and low predictability for switch trials. Once the switch trials appeared, the participants encountered greater conflict with their expectations, and would take longer to solve this conflict, causing longer latencies and greater N2 amplitudes for switch conditions. In other words, switch predictability boosted the original switch costs in the low switch predictability condition. Follow this logic, responses in switch trials should be faster than non-switch trials in the high switch predictability condition. The question arises as to why the present behavioral and ERP results consistently showed no significant difference between switch and non-switch trials in the high switch predictability condition. According to the proactive interference model, activation of the previously used task persists and thus causes either interference with the current task (switch trials) or results in residual activation and thus facilitation (non-switch trials). It meant that in switch trials there would always be interference from the prior trial, which could be diminished, but not abolished. In high switch predictability condition, switch trials prominently outnumbered non-switch trails, which could cause stronger interference than facilitation with the current task. Although higher predictability was achieved in switch trials, the actual effects of switch predictability may be reduced or even eliminated by the interference of the switch mechanism itself.

Language switching could be regarded as a special kind of task switching, given the substantial similarities between them (e.g., Declerck et al., 2017; Stasenko et al., 2017). Thus, the switch predictability effect and the role of the switch mechanism could be considered from the perspective of task switching. Rogers and Monsell (1995) suggested that the ability to switch from one cognitive task to another included two processes, namely endogenous preparation and exogenous regulation (Rogers and Monsell, 1995; Meiran, 1996). Endogenous preparation involves active preparation under expected conditions, including the inhibition of the prior language task set and the activation of the current language task set. In contrast, exogenous regulation is a passive process in which responses need to be adjusted when external stimulation is beyond one’s prediction abilities (Rogers and Monsell, 1995; Sohn et al., 2000). In our opinion, the switch predictability effect on switch costs in the present study was affected by the dynamic influence between active endogenous preparation and passive exogenous regulation. In the low switch predictability condition, the non-switch trials allowed more endogenous preparation and less exogenous regulation, whereas the switch trials were the reverse, leading to longer RTs due to cognitive conflict and the need to adjust the response when faced with the switch trials. In the high switch predictability condition, the switch trials allowed more endogenous preparation and less exogenous regulation, whereas the non-switch trials were the reverse. However, the endogenous advantage for the switch trials in the high switch predictability condition could be eliminated by the switch mechanism when faced with a greater number of switch trials.

### The Process of Bilingual Language Production

According to the language non-specific selection hypothesis, bilingual language production mainly consists of two phases, namely lemma activation and language selection. When the digit was presented, candidate words in the participants’ L1 and L2 were both activated. When the cue was shown, the candidate words competed to be produced. With the modified language switching paradigm in this study, both digit-locked and cue-locked ERPs were examined to reveal the effects of naming language and switch predictability on switch costs in the two different phases.

In the lemma activation phase, the digit-locked analysis revealed neither significant main effects nor interactions in the time windows for 180–280 ms and 350–500 ms post-digit onset. These results suggest that there was no effect of naming language or switch predictability on switch costs during the lemma activation phase. We speculate that switch costs did not occur at the lemma activation phase (Green, 1998; Chang et al., 2016), since no switch signal had yet appeared after a digit’s onset. This is consistent with the findings of Chang et al. (2016), which did not reveal any differences among the naming conditions for the digit-locked ERP data in the stimulus-cue sequence.

In the language selection phase, however, the cue-locked ERP data revealed a different picture. The analysis revealed a negative-going wave, approximately 200 ms after cue onset. This finding was similar to the N2 component reported in previous ERP studies on bilingual language production (Jackson et al., 2001; Christoffels et al., 2007; Guo et al., 2013). And the negative-going ERP that peaked approximately 420 ms after the cue onset may be N400, which reflected the lemma retrieval process (Moreno et al., 2008; Chang et al., 2016). According to the IC model, lemma selection happens after task schema competition in bilingual language production. The language task schema competition stage refers to the phase when L1 or L2 competes to name an object according to the external cue. The lemma selection stage is the process in which the activated candidate lemma competes to be produced. Therefore, it seems that the early negative ERP component corresponds to the language task schema competition stage and the late negative ERP component corresponds to the lemma selection stage.

At the early phase of language selection, i.e., language task schema competition stage, L1-L2 switching direction trials elicited a greater N2 than L2 non-switch trials around 250 ms after onset of the language cue, but no significant difference in N2 amplitude for L1 trials. This indicates a strong effect of naming language on switch costs. Although no independent effects of switch predictability on switch costs were observed during this early phase, it is worth noting that switch predictability and naming language appeared to work together toward switch costs. Particularly, the effect of naming language depended greatly on the levels of switch predictability: in the low switch predictability condition, the N2 component of switch trials was more negative than that of non-switch trials for the L2 only. In contrast, in the high switch predictability condition, there was no such difference in N2 amplitude between switch and non-switch trials for either language. At the late phase of language selection, i.e., lemma selection stage, the cue-locked ERP data did not reveal significant findings. These results provided informative evidence showing that effects of naming language and switch predictability on switch costs mainly occurred in the language selection phase, particularly at the language task schema competition stage. Our result is consistent with Verhoef et al.’s (2010) findings, which found more negative cue-locked ERPs for switch trials than for non-switch trials, suggesting that switch costs were involved in the language task schema competition phase.

## Conclusion

The present study investigated the effects of naming language and switch predictability on switch costs during two phases of bilingual language production, namely the lemma activation phase and the language selection phase. The behavioral results only showed the effects of switch predictability on switch costs, while the ERP results indicated that both naming language and switch predictability affected switch costs, and those effects mainly occurred during the phase of language selection, particularly at the language task schema competition stage.

## Author Contributions

YL, LL, and RW designed the experiments. YL and SC ran the data-collection procedures of the experiments. YL, SC, and RW analyzed and interpreted the data. YL drafted the manuscript. SC, WL, DC, and JZ provided critical revisions of the manuscript.

## Conflict of Interest Statement

The authors declare that the research was conducted in the absence of any commercial or financial relationships that could be construed as a potential conflict of interest.
